# Ameliorating Inflammation in Insulin-resistant Rat Adipose Tissue with Abdominal Massage Regulates SIRT1/NF-κB Signaling

**DOI:** 10.1007/s12013-022-01085-1

**Published:** 2022-07-30

**Authors:** Tianjiao Gao, Shaotao Chen, Yiran Han, Dongmei Zhang, Yi Tan, Yutao He, Mingjun Liu

**Affiliations:** Departments of Acupuncture and Massage, Changchun University of Chinese Medicine, Changchun, Jilin Province, 130117 PR China

**Keywords:** SIRT1, NF-κB, Abdominal Massage, Insulin Resistance, Inflammation, Adipose Tissue, Rat Model

## Abstract

It was the aim of this study to determine whether abdominal massage reverses high-fat diet-induced insulin resistance compared with RSV treatment. A total of sixty male Sprague-Dawley rats were randomly placed in one of four groups:the non-fat diet (NFD), the high-fat diet (HFD), the HFD with abdominal massage (HFD+ AM), and the HFD plus resveratrol (HFD+ RSV). For eight weeks, rats were fed high-fat diets to create insulin resistance, followed by six weeks of either AM or RSV. Molecular mechanisms of adipogenesis and cytokine production in rats with high-fat diets were investigated. The model rat adipose tissue showed significant improvements in obesity, glucose intolerance, and the accumulation of lipid in the body [the total cholesterol level (TC), triglycerides (TG), high-density lipoprotein cholesterol (HDL-C), and low-density lipoprotein cholesterol (LDL-C)], metabolic effects of glucose [The fasting blood glucose (FBG), Fasting insulin levels (FINS)], inflammatory status [interleukin-6 (IL-6) and tumor necrosis factor (TNF)-α, C-reactive protein (CRP)], and macrophage polarization after AM or RSV treatment. Further, AM increased SIRT1/NF-κB signaling in rat adipose tissue. Accordingly, in rat adipose tissue, our results indicate that AM regulates the secretion of proinflammatory cytokines, blood sugar levels, and related signaling pathways, contributing to improvement of IR, which may serves as a new therapeutic approach for the treatment for IR.

## Introduction

Insulin resistance (IR) is the greatest potential threat to human health [1]. An IR is a condition in which peripheral insulin-target organs, such as muscle, liver, and adipose tissue, are less sensitive to normal levels of insulin, leading to a decrease in the efficiency of glucose uptake and utilization in the blood, an increase in pancreatic insulin secretion and eventually IR [2]. Metabolic syndrome and type II diabetes are more likely to develop when IR is prevalent. Further, obesity-induced inflammation contributes significantly to IR [3].

Overeating causes extra energy to be stored as triglycerides, which can be deposited in ectopically situated tissues such the liver, muscle, and placenta, resulting in obesity. A dense network of capillaries of infiltrates adipose tissue, particularly visceral fat, and macrophages and lymphocytes inhabits it. Using this construct, a sustained, dynamic cross-talk is established between energy metabolism and the immune response, allowing for communi-cation with other insulin target tissues and organs [4]. Studies have shown how many macrophages are involved in the adipose tissue recruitment, which is a reservoir of inflammatory factors [5].

A lack of effective treatment options exists for IR-related diseases in modern medicine.

A number of lifestyle interventions, like quitting smoking, exercising and making dietary changes, can result in weight loss, lower blood glucose levels, lower blood pressure, and regulate lipid metabolism disorders. Both patients and clinicians often experience the side effects of western medicine, as well as long-term low efficacy or the lack of efficacy [6–9]. Thus, clinical therapies that are safe, effective, and easy to use are urgently needed to treat IR.

A variety of substrates, including histones and non-histones, are deacetylated by Sirtuin 1 (SIRT1) [10–12]. Inflammatory responses are triggered by nuclear factor-kappa B (NF-κB), a large number of lysine sites on NF-κB’s component p65 may be acetylated, stimulating the transcription of NF-κB and its capacity to bind to target genes’ promoter regions to drive the inflammatory response [13–16]. SIRT1 reduces NF-κB acetylation at lysine 310 (K310) via direct interaction with acetylation of NF-κB, inhibiting its transcriptional activity and decreasing the production of proinflammatory genes [17–20]. These suggest that NF-κB–mediated inflammatory genes are associated with SIRT1, which can suppress inflammation by regulating NF-κB acetylation.

As a result, we hypothesized that abdominal massage may act to reduce chronic inflammation in insulin-resistant obese patients by regulating SIRT1 expression in adipose tissue. It is possible that other inflammatory signaling pathways could achieve the same effects if SIRT1 is responsible for deacetylation of NF-κB.

As an external remedy, a massage utilizing traditional Chinese medicine is commonly used in clinical settings. “External Tong meridians and internal regulating Zang Fu organs” are the benefits of the abdominal massage (AM) method, which refers to a gentle massage of the abdomen to treat various diseases, including overweight and metabolic syndrome [21]. In the clinical treatment of patients with obesity who is taking insulin alone, AM has been demonstrated to improve triglyceride metabolism in the body [22]. The AM treatment significantly increased SIRT1 mRNA and protein levels in skeletal muscle via PGC-1, as reported previously [23]. This research examined AM’s effects on an IR rat model induced by a high-fat diet and compared them to the effects of the SIRT1 agonist resveratrol (RSV) for the purpose of better understanding the relationship between them.

## Research Materials and Methods

### Establishing Animal Models and Grouping Them

At Changchun University of Traditional Chinese Medicine, all animal experiments were conducted according to the institution’s animal care guidelines and the Animal Experimental Ethics Committee has approved this experiment (approval NO. 2021171). Sprague-Dawley rats weighing 200 ± 20 g were used in our study, which involved sixty 8-week-old male rats, and were supplied by Changchun Yisi Laboratory Animal Technology Co., Ltd. In the Changchun University of Chinese Medicine’s specific pathogen-free animal housing, all rats were housed (animal facilities licensed under the following number: syxk (Ji) 2018-0014) under temperature-controlled (20–25 °C) conditions with 45% relative humidity and rhythmic light (12 to 12 h). Daily, the bedding and water were changed. A supply of bedding and feed materials was provided by Changchun University of Chinese Medicine’s Experimental Animal Center. Prior to this experiment, the animals were allowed a week to acclimate.

In adaptogenic feeding, we randomly assigned 15 rats to a non-fat diet (NFD) group and 45 rats to a high-fat diet (HFD) group for one week each. In the NFD group, rats were supplied with a basic chow diet (3.8 kcal/g, consisting of 70% carbohydrates, 20% proteins, and 10% fat). In the HFD group, rats received 5.4 kcal/g of carbohydrates, 15% of protein, and 46.5% of fat in their diet [24]. During the 8 weeks of feeding, we conducted the glucose clamp test on 45 rats from the HFD group to determine the glucose infusion rate. Each group of rats was randomly divided into three groups: the high-fat diet (HFD) group, the abdominal massage(AM) group, and the resveratrol (RSV) group (*n* = 15 per group). As controls, 15 rats from the NFD group were used.

### Treatments

Operators were trained to utilize the TAP-II Massage handgrip strength apparatus (Shanghai The Yilian Medical Development Company, Inc.) prior to treatment to acquire the proper massage technique and pressure application (instrument weight: 0.5 kg). As part of the AM group, each rat received friction and massage once a day for six weeks, together with acupressure (RN4 Guanyuan, RN6 Qihai, RN12 Zhongwan, ST25 Tianshu) [25]. In “the Appendix for Experimental Acupuncture”, acupoint positions are described. One daily dose of 200 mg/kg RSV (r817263; Macklin Company, Shanghai, China) was administered orally once a day to rats in the RSV group for 6 weeks. A 6-week period was spent giving the NFD and HFD groups equal amounts of normal saline orally. Lee’s index was calculated every week by measuring the body mass, food consumption, and nasal length of each rat. The formula is: (weight × 1000)^1/3^/body length (cm). Each rat was anesthetized for blood sampling, which was followed by collection of liver and adipose tissue, which was stored in a freezer at a temperature of –80 °C until analysis.

### An Analysis of Biochemical Indices

Biochemical kit (Rayto Life and Analytical Sciences Co., Ltd., Shenzhen, China) were used to deteimine serum levels of total cholesterol (TC), triglycerides (TG), high-density lipoprotein cholesterol (HDL-C) and low-density lipoprotein cholesterol (LDL-C). A glucose oxidase test was performed to determine fasting blood glucose levels (FBG). With a kit for ELISA (enzyme-linked immunosorbent assay), fasting insulin (FINS), inflammatory cytokines, including interleukin-6 (IL-6) and tumor necrosis factor (TNF)-α, as well as C-reactive protein (CRP) were measured in peripheral blood. Calculated by combining FBG×FINS/22.5, insulin resistance in the body is measured by homeostasis modeling assessment-estimated insulin resistance (HOMA-IR).

### Western Blotting

Radiation immunoprecipitation assay lysis buffer (PC102; Shanghai Yazyme Biomedical Technology Co., Ltd., Shanghai, China) was used to obtain the total protein content from the adipose tissue and by using bichromoninic acid, the protein concentration could be determined. In this experiment, a “separating gel” and a “concentrating gel” were prepared using Gel Fast Preparation Kit for polyacrylamide gel electrophoresis. Polyacrylamide gels containing 8% sodium dodecyl sulfate were used for the separation of proteins (30 mg), which were electroblotted onto PVDF membranes. In TBST (Tris-buffered saline with tween) 5% nonfat milk was applied for an hour to PVDF membranes at room temperature, and Incubation of the primary antibodies (1:500) overnight at 4 °C on a shaking plate: The sirtuin 1 (600-401-gw4), the NF-κB (100-4165) primary antibodies available from Rockland (Philadelphia, USA) and β-actin (W16197A) primary antibody (Biolegend, San Diego, CA, USA) were used as loading controls.

A goat anti-rabbit secondary antibody (1:5000; S0001, Affinity Biosciences, Cincinnati, OH, USA) conjugated with horseradish peroxidase was incubated with the membrane after TBST washes and shaking. After washing the PVDF membrane with TBST, a chemiluminescent blot was developed with electrochemical detection. An imaging system (Bio-Rad, 1708370) that uses chemiluminescence was used to determine the results. Gel Pro software was used to evaluate the densitometric values of the bands of interest in order to quantify the expression level of the protein of interest in each sample as a ratio of the protein of interest’s optical density to that of the internal reference protein (β-actin).

### Fluorescence-activated Cell Sorting (FACS)

Adipocyte adipose tissue obtained from epididymis fat cells was collected after extensive trituration in phosphate-buffered saline solution pre-chilled to stop cell growth, and an even suspension of a single cell was pipetted. In aliquots of 1.0 × 10^6^ cells, ten microliters of the cell suspension was removed and diluted 1000-fold to get a trypan blue stain, and counted under a microscope. To a blend of 1.25 μL PE anti-mouse CD11c antibody (117307, Biolegend, San Diego, CA USA), 2 μL FITC anti-mouse F4/80 recombinant antibody (157309, Biolegend, San Diego, CA USA), and 2.5 μL APC anti-mouse CD206 antibody (141707, Biolegend, San Diego, CA USA), fluorescently labeled CD45 antibody was added (CD45 anti-perCP antibody in the form of 103129 from Biolegend, San Diego, USA) and a 30 minute incubation at 4 °C in the dark was conducted. Centrifugation was performed after adding 2 mL of diluted buffer to the cells, and the supernatant was discarded after washing the cells. We resuspended the cells in Flow Cytometry Staining Buffer (500 μL) and then detected them with the FACS system. A CD45^+^F4/80^+^CD206^+^ macrophage was identified as M2 and a CD45^+^F4/80^+^CD11c^+^ macrophage as M1.

### Analyses of Statistical Data

SPSS 22.0 and GraphPad Prism 8.0 were used to carry out the statistical analysis. We present the data in the form of mean ± standard deviation. The comparisons between groups were based on the two-tailed Student’s *t* test, with *p* values less than 0.05 considered statistically significant.

## Results

### Biochemical Changes in Rats with IR Induced by HFD

**Fig. 1:**
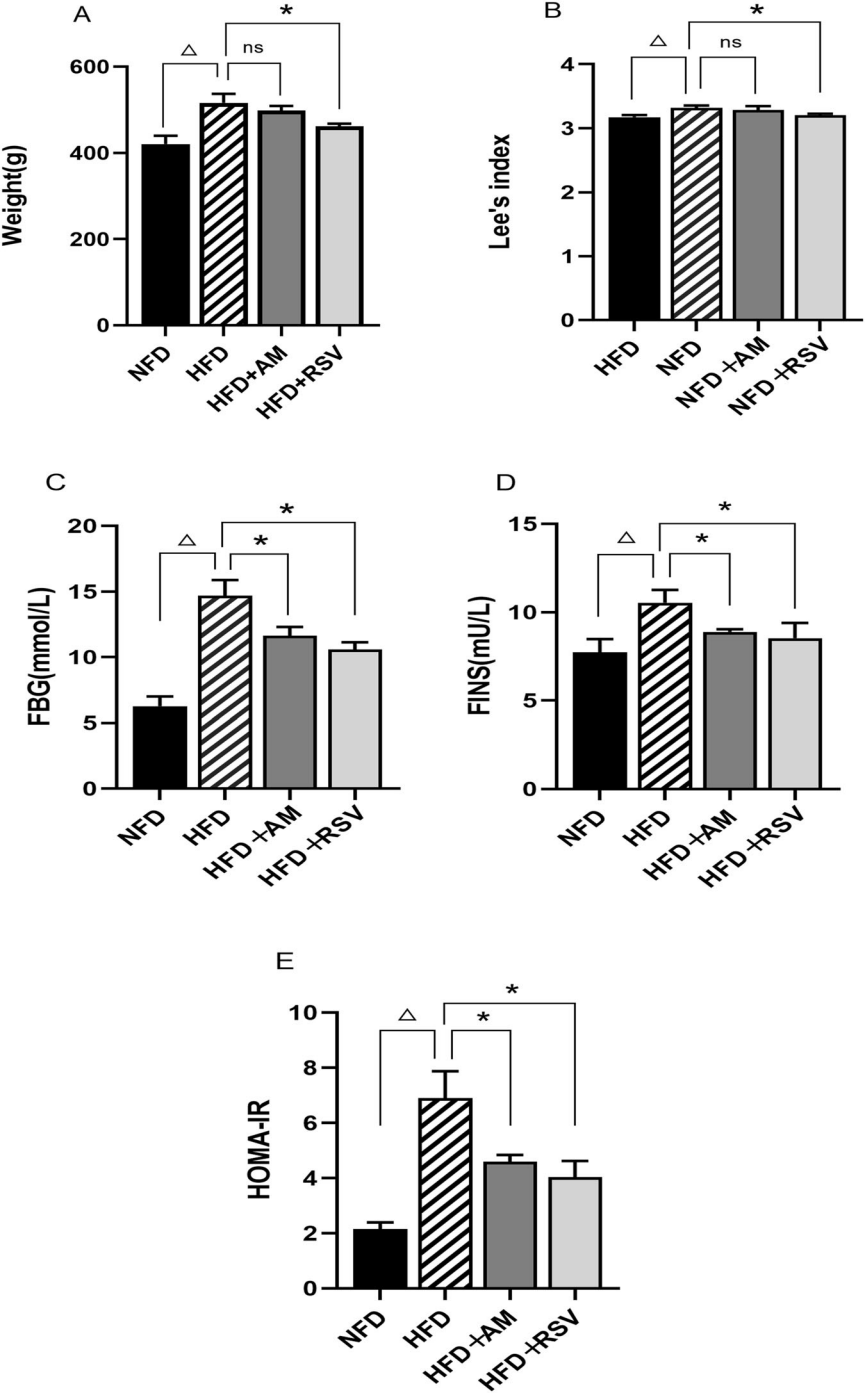
Rats on HFD feed are relieved of IR by AM. **A** Weight, **B** Lee’s index, **C** FBG, **D** FINS, **E** HOMA-IR. Statistically significant differences with NFD groups were at ^Δ^*p* < 0.05, and significant differences with HFD groups were at *p < 0.05 (NFD diet with normal fat, HFD fat-rich diet, RSV resveratrol, AM abdominal massage)

In this study, we evaluated the general characteristics of HFD-induced IR rats receiving AM and RSV treatments(Fig. 1). Utilizing Lee’s index and HOMA-IR, the severity of symptoms was assessed. In contrast to the NFD group, significantly more body weight was achieved in the HFD group (*p* < 0.05). Comparing the HFD + RSV group to the HFD group, the body weight of the HFD + RSV group was significantly lower (*p* < 0.05). Although it was true that body weight decreased in the HFD + AM group, no statistical significance was found. With respect to Lee’s index, the HFD + AM and HFD + RSV groups had significantly lower values than the HFD group (*p* < 0.05). Six weeks after receiving treatment, the rats in the HFD group had significantly higher HOMA-IR levels.

### An Investigation of the Effects of AM on Lipid Metabolism in HFD-induced IR Rats

**Fig. 2:**
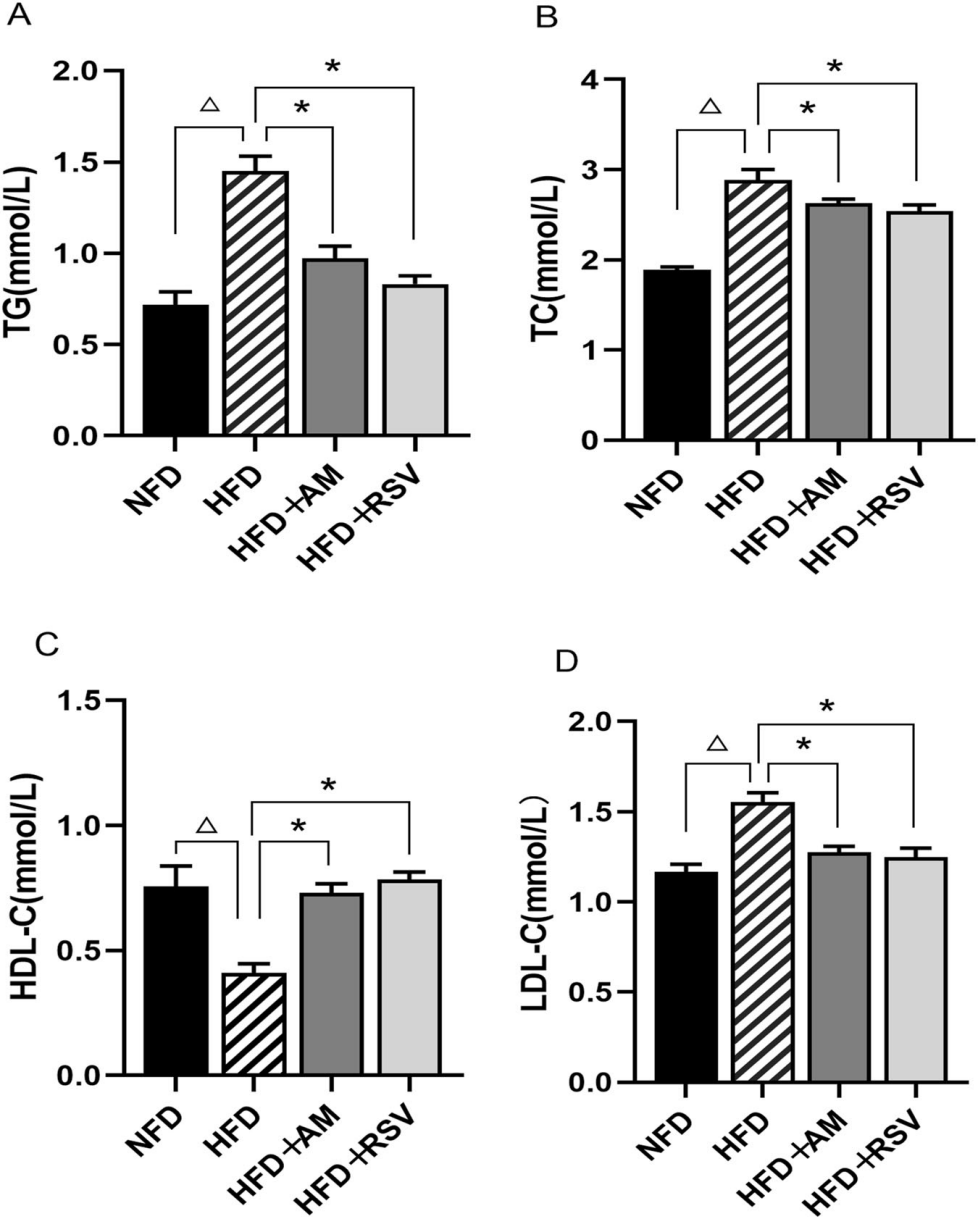
AM’s effect on lipid metabolism in HFD-induced insulin resistance rats. **A** TG, **B** TC, **C** HDL-C, **D** LDL-C. Statistically significant differences with NFD groups were at ^△^*p* < 0.05, and significant differences with HFD groups were at *p < 0.05 (NFD diet with normal fat, HFD fat-rich diet, RSV resveratrol, AM abdominal massage)

A comparison of the serum levels of TG, TC, and LDL-C between the HFD and NFD groups after 14 weeks of feeding shows that the HFD group’s levels were higher (*p* < 0.05). There was a significant difference in HDL-C levels between the HFD and NFD groups as well (*p* < 0.05). Compared to the HFD group, the HFD + AM and HFD + RSV groups showed markedly lower levels of lipid accumulation (Fig. 2).

### HFD-induced IR Rats receiving AM Exhibit Reduced Inflammatory Responses

**Fig. 3:**
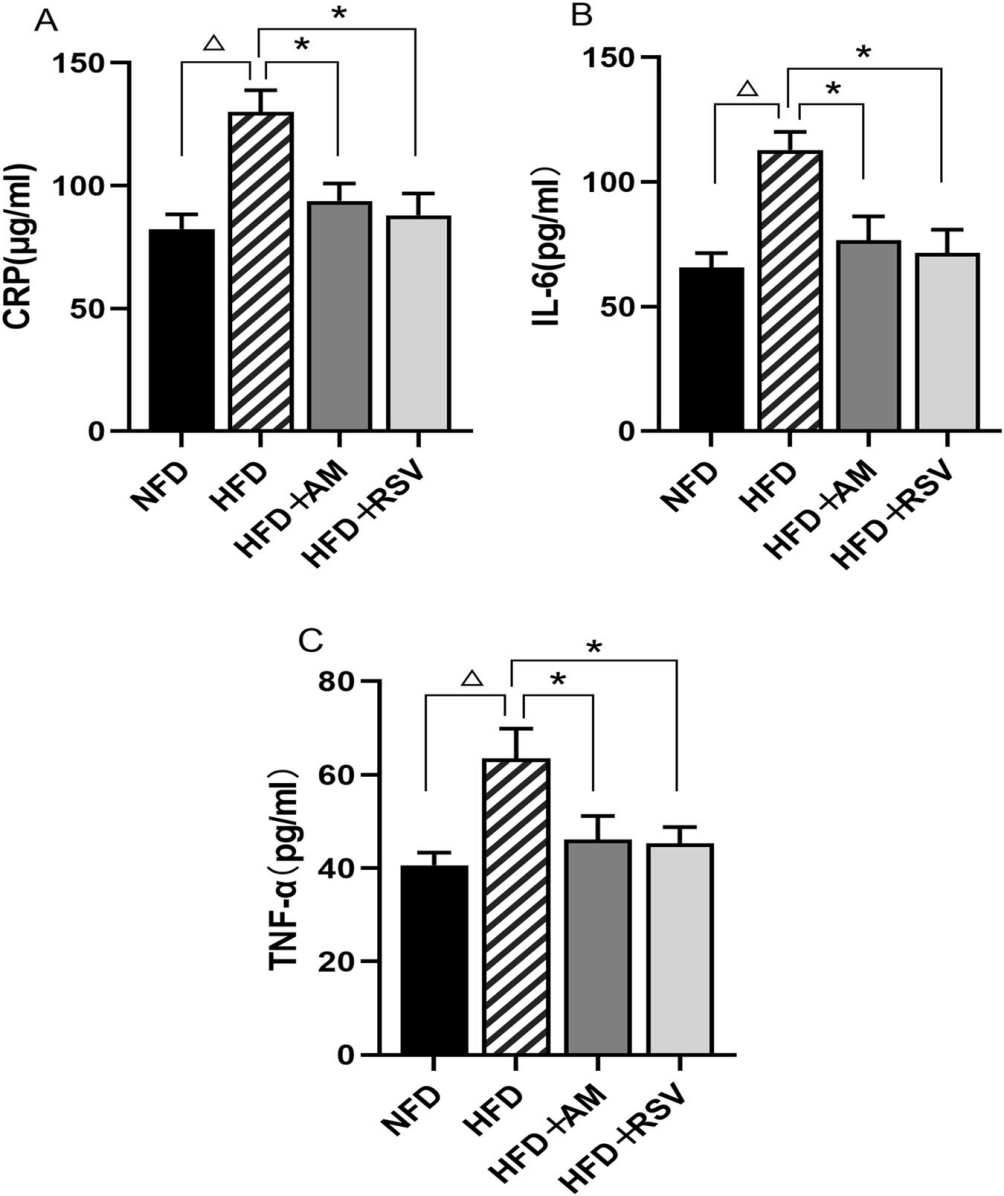
AM Attenuates the inflammatory state of the body in IR rats induced by HFD. **A** CRP, **B** IL-6, **C** TNF-α. Statistically significant differences with NFD groups were at △*p* < 0.05, and significant differences with HFD groups were at *p < 0.05. (NFD diet with normal fat, HFD fat-rich diet, RSV resveratrol, AM abdominal massage)

Using an ELISA kit, we examined the levels of pro-inflammatory cytokines (TNF-α and IL-6) as well as CRP in the serum of rats. In comparison to NFD rats, HFD rats had considerably greater levels of CRP in their serum, as seen in Fig. 3A. (*p* < 0.05). The serum CRP levels of HFD + AM rats reduced considerably after AM intervention compared to those of HFD rats, indicating that AM may effectively relieve inflammatory CRP levels in the blood of IR rats induced by HFD. HFD + RSV rats had substantially lower serum CRP levels than HFD rats (*p* < 0.05), showing that SIRT1 activation by RSV may lower CRP levels in the blood of HFD-induced IR rats and, as a result, reduce inflammation. TNF-α and IL-6 levels were significantly elevated in HFD rats compared with NFD rats (*p* < 0.05), as depicted in Fig. 3B and C. Following treatment with AM or RSV for six weeks, IL-6 and TNF-α levels in serum of HFD + AM and HFD + RSV rats dropped significantly compared with those of HFD-induced IR rats (*p* < 0.05).

### The Activation of the SIRT1/NF-κB Pathway by AM Ameliorates Inflammation in the Adipose Tissue of Rats Induced by HFD

**Fig. 4:**
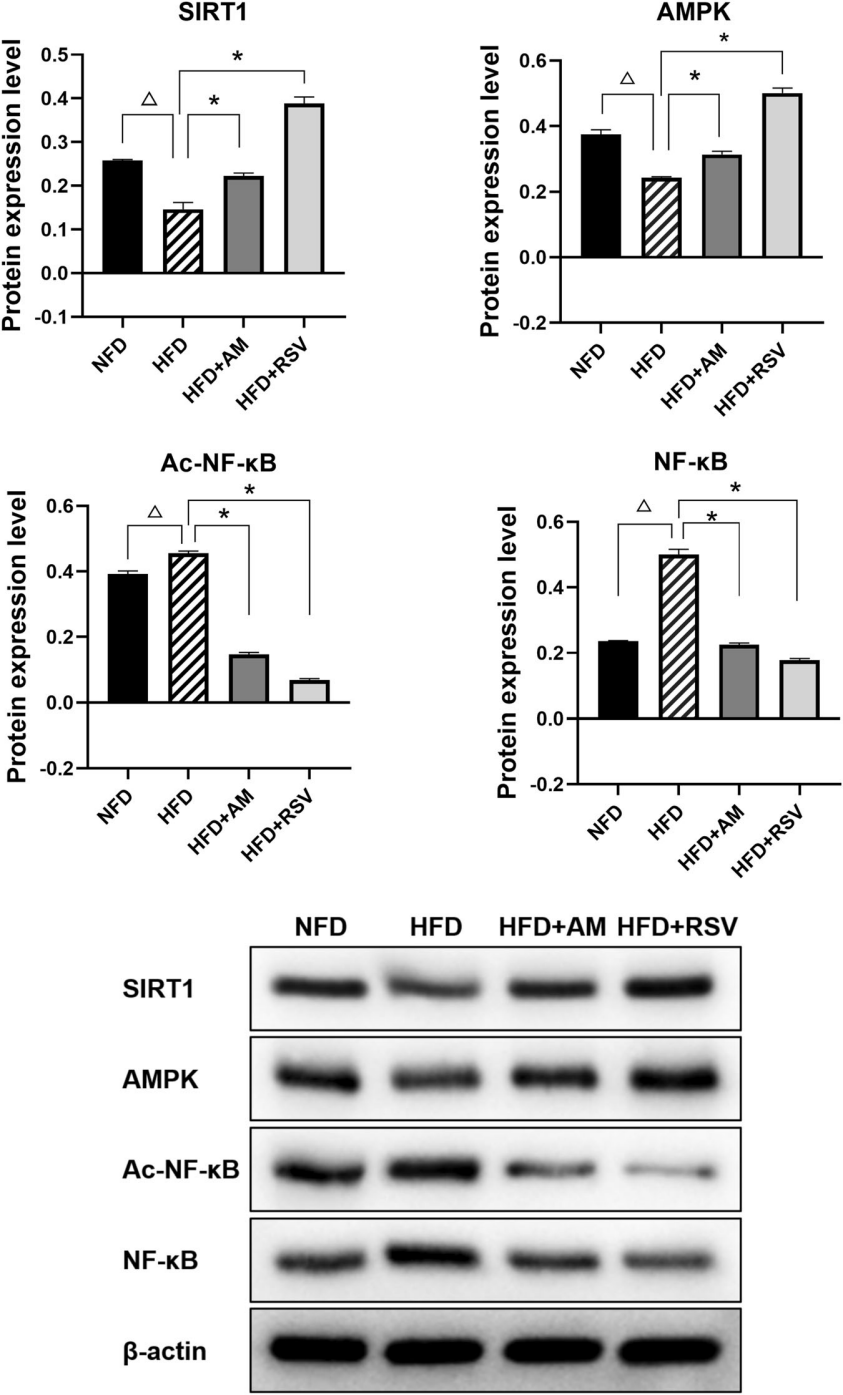
AM activation of the SIRT1/NF-κB pathway reduces inflammation in adipose tissue of rats subjected to HFD induced insulin resistance. Here are the protein expression levels for SIRT1 and NF-κB in adipose tissues of insulin-resistant rats induced by high-fat diets. Statistically significant differences with NFD groups were at ^Δ^*p* < 0.05, and significant differences with HFD groups were at *p < 0.05. (NFD:Diet with normal fat, HFD:Fat-rich diet, RSV:resveratrol, AM:abdominal massage)

We then looked at how AM affected SIRT1 regulation in the adipose tissue of HFD-induced IR rats (Fig. 4). The HFD group showed a significant decline in SIRT1 and AMPK expression in the adipose tissue compared to that of the NFD group (*p* < 0.05), while a significant increase in ac-NFκB levels was observed in the adipose tissue of the HFD group compared with the NFD group (*p* < 0.05). The finding illustrates that NF-κB acetylation is altered in adipose tissue when there is inflammation.

However, after continuous AM or RSV intervention, SIRT1 and AMPK levels were significantly increased compared with those in NFD rats (*p* < 0.05), the presence of SIRT1 and AMPK levels was significantly increased after continuous AM or RSV treatment in comparison with NFD rats, while Ac-NFκB levels were markedly reduced (*p* < 0.05).

### Activated Macrophages and Polarized Macrophages in IR Rats Induced by HFD

**Fig. 5:**
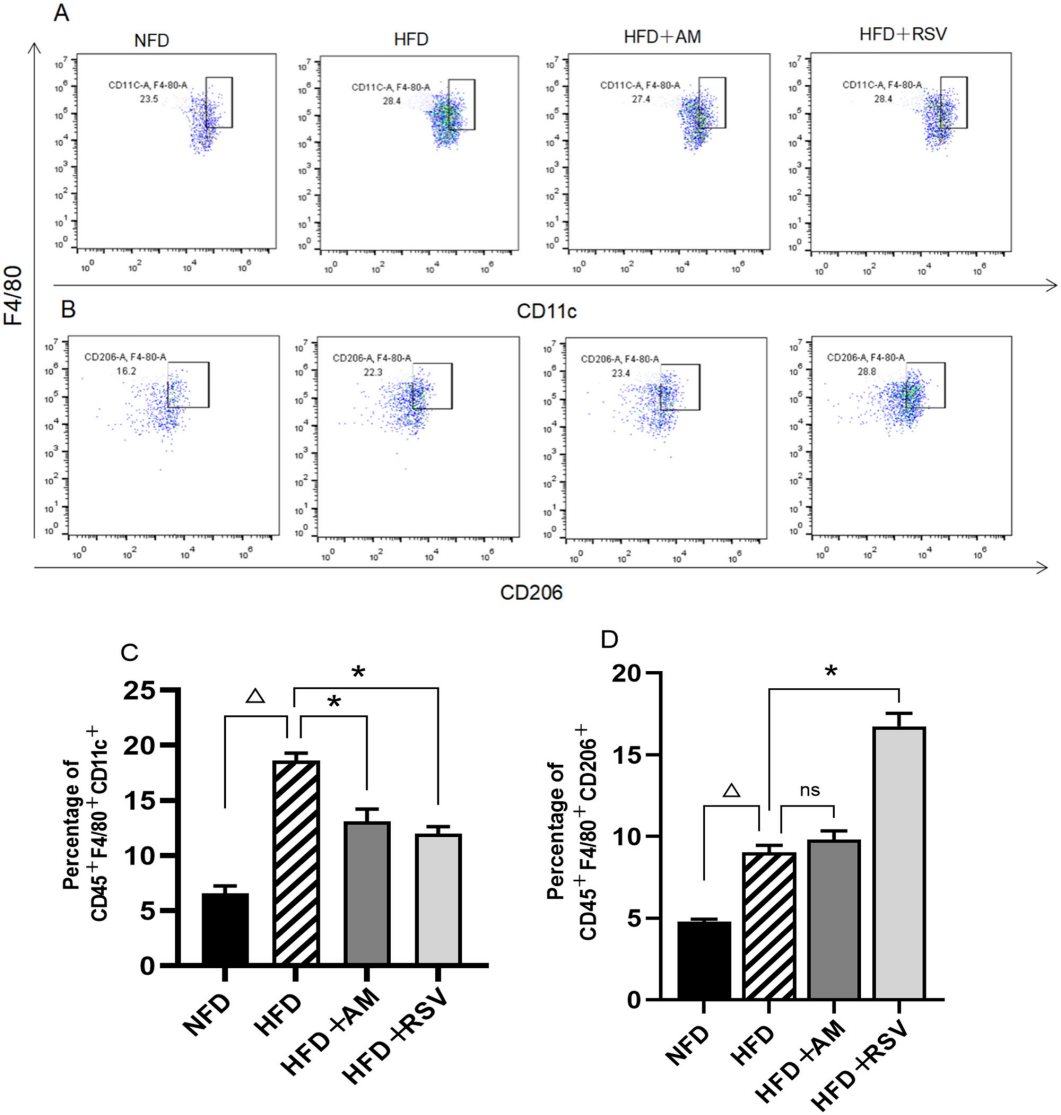
Effects of AM on macrophage polarization in HFD-induced IR rats. **A** Representative flow pictures of M1 type macrophage, **B** Representative flow pictures of M2 type macrophages, **C** M1 type macrophage proportion in eWAT, **D** M2 type macrophage proportion in eWAT. Statistically significant differences with NFD groups were at ^Δ^*p* < 0.05, and significant differences with HFD groups were at *p < 0.05. (NFD:Diet with normal fat, HFD:Fat-rich diet, RSV:resveratrol, AM:abdominal massage)

After AM and RSV intervention, Flow Cymetry was used to measure changes in the relative proportions of M2 (CD45^+^F4/80^+^CD206^+^) and (CD45^+^F4/80^+^CD11c^+^) macrophages in the epidydimal white adipose tissue (eWAT). In contrast to NFD rats, rats induced with HFD displayed a markedly increased number of M1 types of macrophages and a concurrent increase in M2 types of macrophages (Fig. 5). When compared to that of the HFD group, both AM and RSV were able to reduce the M1-type macrophage proportion in the eWAT of HFD-induced IR rats (*p* < 0.05). Rats in the HFD + RSV group, however, displayed a significant increase in M2-type macrophages (*p* < 0.05). Additionally, AM increased the proportion of M2 macrophages, but this was not statistically significant (*p* > 0.05). AM and RSV reduced inflammation in the eWAT by modulating macrophage polarization to the M2 type.

## Discussion

Inflammation from insulin-resistant obesity is a leading cause of several metabolic disorders, including type 2 diabetes and atherosclerosis [26–28]. Pharmacological and medicinal research has dedicated significant resources to elucidating the mechanism of IR due to the lack of effective treatment methods. As far as we are aware, this is the first study to look at AM’s effect on inflammation in rats with IR induced by HFD. Both AM and RSV appeared to influence HFD-induced IR and inflammation in the present study. The findings of this study were consistent with those reported in previous studies [23, 29]. This study utilized biochemical kits to measure adipocytokine levels and ELISA kits to measure proinflammatory cytokines. HDL-C, LDL-C, TC, TG, CRP, TNF-α, and IL-6 levels were abnormal in the HFD group, indicating harm to lipid metabolism and inflammation after a HFD-induced insulin resistance diet. Nevertheless, HFD-induced IR rats showed positive regulation of relevant proinflammatory cytokines and adipocytokines due to AM, showing that AM improved inflammatory conditions and lipid metabolism disorders.

The paracrine effects of IL-6, TNF-α, and IL-1β in the eWAT, as well as that induced by macrophage-secreted substances, may be involved in inflammatory signaling within insulin target cells, inhibition of B kinase (IKK), Jun-terminal kinase (JNK), and other serine kinases are activated as a consequence [30]. Signaling by JNK1 and IKK are significantly increased in mouse skeletal muscle, fat, and other tissues during the IR state. These serine kinases can activate target transcription factors including AP-1 and NF-B, causing the transcription of a variety of inflammatory pathway genes [31, 32]. SIRT1 deacetylation can directly modify the K310 site of p65, lowering its acetylation level and inhibiting transcriptional activity. NF-κB is a SIRT1 substrate, and SIRT1 deacetylation can directly modify the K310 site of p65, reducing its acetylation level and inhibiting transcriptional activity. AMPK, on the other hand, lowers lipid buildup by lowering blood glucose and lipid acid levels, enhancing insulin sensitivity [33–36]. In a prior study, we discovered that AM controls inflammation in diabetic insulin-resistant rats through mediating SIRT1 deacetylation via NF-κB p65 (K310). As a result, we hypothesized that AM-activated SIRT1 would deacetylate NF-κB in the eWAT and modulate inflammatory factor transcription. Using western blot analysis, we monitored the gene and protein expression of SIRT1, AMPK, and ac-NFκB in the eWAT. The results indicated that AM modulated SIRT1 expression to stimulate the NF-κB signaling pathway in the eWAT and lowering NF-κB acetylation, which was consistent with our hypothesis.

It is incredible how adaptable macrophages are, a type of immune cell that contributes to both humoral and cellular immunity [37, 38]. Mature macrophages can polarize to respond to changes in the environment when the local environment is activated. Macrophages can be categorized into M1 and M2 kinds based on various cell-surface markers after polarization, as well as diverse roles [39, 40]. The M1 macrophage plays a role in the beginning of the inflammatory response by releasing pro-inflammatory factors like IL-6 and TNF-α, it also phagocytoses, eliminates invading microorganisms, and inhibits cancer’s escape from the immune system.Macrophages of the M2 type can secrete anti-inflammatory molecules such as IL-10 and IL-4 [41, 42]. As M1 and M2 macrophages are distinct subsets of macrophages that affect inflammation differently, they may contribute significantly to a wide range of inflammation-related conditions, and inflammation is thought to be regulated by the macrophage program [43]. In the event of macrophage activation, chemokines are released, which promotes macrophage recruitment, increasing macrophage content can promote chronic inflammation and ultimately contribute to IR.

By using flow cytometry, we determined the number of macrophages in the eWAT of rats exposed to HFD-induced IR. We found that rats in the HFD group displayed higher levels of proinflammatory factors than those in the NFD group, indicating that the presence of proinflammatory factors in the eWAT correlates with the pathological changes. This suggests that macrophage polarization in the adipose tissue favors M1-type macrophages, causing an imbalance between proinflammatory and antiinflammatory responses. IR rats treated with AM showed an increase in M2-type macrophages, indicating that macrophage polarization toward the M2 type has occurred in adipose tissue. Accordingly, AM may exert therapeutic effects on rats with IR induced by HFD by modulating macrophage polarization in eWAT.

It is recognized that massage therapy represents a method of Chinese medicine and external treatment that can increase energy metabolism and encourage oxidative utilization of body fat [44, 45]. Additionally, massage stimulates blood circulation to adipose tissue, reduces appetite and promotes gastrointestinal motility. AM manipulation has the advantage of making treatment easy with excellent amplitude and strength, concentrating the site of treatment, and making the process efficient and atraumatic, thus improving patient comfort and clinical efficacy. Given impediments of the current trial, further investigations of the impact of AM treatment on aggravation in different tissues of the insulin-resistant obese rat model are required, which will be enlightening for accuracy treatment in the facility.

To be more precise, pertinent measurements should be made sequentially in various organ groups, such as the pancreas, intestine, and brain, we aim to improve our understanding of the mechanisms behind AM’s action in IR obesity by achieving a more comprehensive study.

## Conclusion

Summing up, SIRT1 has a significant impact on energy metabolism, and NF-κB signaling is involved in inflammation, which is critical for obesity and insulin resistance. By regulating the expression of SIRT1, the AM method can inhibit inflammation and control IR, therefore, acetylation of NF-κB is reduced, polarization status of macrophages is altered, and NF-κB signaling in adipose tissue is activated. Research from this study provides insights into developing AM as a potential therapeutic option for IR-associated metabolic and inflammatory factors secretion problems. However, NF-κB was examined only as a SIRT1-

related substrate in this experiment; therefore, it remains to be determined whether AM controls SIRT1 to regulate other inflammatory signaling pathways like AP-1. In this regard, further research is necessary to be able to determine the potential clinical application of AM in treating obesity with IR.
